# Network analysis of assistive technology stakeholders in Malawi

**DOI:** 10.1080/16549716.2021.2014046

**Published:** 2022-02-02

**Authors:** Emma M. Smith, Ikenna D. Ebuenyi, Juba Kafumba, Monica Jamali-Phiri, Alister Munthali, Malcolm MacLachlan

**Affiliations:** a Assisting Living and Learning Institute, John Hume Building, Maynooth University, Maynooth, Ireland; b Centre for Social Research, Chancellor’s College, University of Malawi, Zomba, Malawi

**Keywords:** Self-help devices, low and middle income countries, assistive products, health policy, policy development

## Abstract

**Background:**

Assistive technologies promote participation and quality of life for people with disabilities and other functional limitations. There is a global call to develop and implement policies to improve access to assistive technologies. In response, a stakeholder led initiative in Malawi is working towards the development of such a policy.

**Objective:**

The objective of this study was to assess the existing network of stakeholders, and the strength of relationship between organizations who deliver assistive products and related services.

**Method:**

We conducted a survey-based network analysis of assistive technology stakeholder organizations in Malawi.

**Results:**

Stakeholders (n = 19) reported a range of connections, from no awareness to collaboration with organizations within the assistive technology network. No single organization or government ministry was most central to the network. International NGOs were less central to the network than local organizations for disabled people, service providers, and ministries.

**Conclusion:**

The assistive technology stakeholder network in Malawi is distributed, with a range of responsibility across a variety of stakeholders, including three government ministries. An effective assistive technology policy must engage all stakeholders and may benefit from a collective leadership approach that spans the inter-sectoral need for a cohesive assistive technology system.

## Background

Assistive products are devices or tools which are intended to promote independence and participation for individuals with disability or functional limitation associated with illness, injury, ageing or other chronic conditions [1]. Assistive technology is a generic terms and represents the products and related services which enable people to improve their functioning, participate in society, and promote well-being [2,3]. Over one billion individuals in the world are currently in need of some sort of assistive technology, and this number is expected to exceed 2 billion by 2050 [4]. However, current estimates suggest only 1 in 10 persons who require assistive technologies have access to them [4]. This number may be even lower in some lower resourced environments. For example, in Malawi, research suggests only 4.5% of the total population of persons with disabilities have access to or use an assistive product [5].

The importance of assistive technology is enshrined within the United Nations Convention on the Rights of Persons with Disabilities [6], and is more explicitly identified in the World Health Assembly Resolution EB142.R6, ‘Improving Access to Assistive Technology,’ which was passed on the 15^th^ of March, 2018 [7]. This resolution called on member states to develop policies and systems which align with a priority assistive products list developed by the World Health Organization’s Global Cooperation on Assistive Technology (GATE) initiative, to improve access to assistive technology globally [7]. Assistive products are also recognized as crucial mediators and moderators for the equitable achievement of the SDGs; leaving no-one behind [8].

In response to this resolution, and in alignment with the Malawi National Disability Mainstreaming Strategy [9], the Assistive Product List Implementation Creating Enablement of Inclusive SGDs (APPLICABLE) project was developed and launched in Malawi on 6 December 2019 [10]. This project is an action-research approach to stakeholder-led policy development and implementation [11]. The approach of the APPLICABLE project recognizes the complexity of assistive technology provision at the national level, and the necessary engagement of multiple ministries, direct service providers, organizations of persons with disability (OPDs) and local non-governmental organizations, as well as development partners and international non-governmental organizations (NGOs) [12,13].

This inherent complexity, with multiple stakeholders hold responsibility for the policies and their implementation, presents a challenge for policy development [12,13]. Therefore, at the outset of the APPLICABLE project, the team identified a need to understand the existing network of organizations engaged in assistive technology provision in Malawi, to help identify those who would be best placed to both oversee and implement policies which were developed. Developing this understanding is critical to identifying ministries who may act as policy holders, and the nature of their relationship with the broader stakeholder community. The objective of this study was to understand and measure the strength of network of the existing assistive technology related organizations in Malawi.

## Methods

We conducted a cross-sectional network analysis. Data were collected using Qualtrics survey software [14]. This research was conducted as part of a larger project to develop an assistive technology policy, therefore the organizations included in this study were each previously engaged as part of this initiative. A list of organizations who are currently providing access to assistive technology or assistive technology services in Malawi (n = 30) was developed by the research team and verified in a stakeholder workshop at the outset of the larger project. This list was used to distribute the survey to relevant organizations for the network analysis.

Each organization was asked to answer demographic questions to categorize their organization, in addition to questions regarding network connections and the strength of those connections. Each organization was asked to ‘Please rate the current relationship between your organization, and each of the organizations listed below,’ and provided with a list of organizations currently active in delivering assistive technology products or services, as determined by the research team, based on current and previous research, and local knowledge. Organizations were also given the opportunity to list other key stakeholders whom they engaged with in their work on a regular basis. Organizations were asked to rate the strength of each relationship on a five-point scale. Table 1 outlines each point in the scale, the definition provided to respondents in the survey, and the weighting assigned to each for analysis.

**Table 1.** Description of survey scale

Strength	Weight	Definition Provided
No Relationship	0	I am not aware of the work this organization is doing
Awareness	1	We are aware of the work done by this organization, but our work is entirely independent.
Communication	2	Our organization actively shares information with this organization as we work towards our own goals. We do not currently cooperate or collaborate on any initiatives.
Cooperation	3	Our organization actively shares information and sometimes has shared activities (less than three times iai year). Referral of clients is included in this category.
Collaboration	4	Our organization actively shares information, and frequently has shared activities (more than 3 times a year). We plan and work together towards shared goals, projects, and initiatives.

### Analysis

We used NodeXL for both network metric calculations and graphical representation of the network. We calculated a variety of network metrics to demonstrate the strength of the network. The term *indegree* represents the number of connections reported about an organization by others, whereas the term *outdegree* represents the number of connections to other organizations reported by an organization itself. Weighted indegree and outdegree are also presented and weighted by the strength of inward or outward connections. This was calculated as the sum of weights of each connection. *Betweenness centrality* is the degree to which the organization is at the ‘centre’ of the network, which can also be thought of as the ability of an organization to link other organization together.

We also completed a graphical analysis of the network. This graph places organizations which are more central to the network at the centre of the graph, and those which are more peripheral at the outer edges of the graph. Line width is determined by strength of connection, and lines are directional, indicated by the arrow-head at the end of the line. In cases where lines are unidirectional, this indicates the relationship was identified by only one of the two stakeholders concerned. In the case of bidirectional relationships, where both of the stakeholders indicated a relationship with each other, arrows are found at both ends of the line. Individual organizations are represented by shape/coloured nodes. Organizations closer to the centre have more connections (in and out) to those around them than organizations nearer to the edge.

## Results

### Respondent organizations

A total of 19 organizations participated in the network analysis, including four government ministries, one service provider, six OPDs, and eight international non-governmental organizations (NGOs). This represents a response rate of 63.3%. Results include connections between these 19 organizations, and do not extend to organizations who did not participate. Further, there were no organizations identified in the ‘other’ category by respondents. Table 2 outlines basic demographic information for the respondent organizations. Total number of respondents for each category may be higher than 19 as respondents were able to endorse more than one category.

**Table 2.** Demographic information for respondent organizations

Factor		# Respondents
Disability Focus	Physical	6
	Intellectual	1
	Psychosocial	1
	Developmental	4
	Sensory	3
	All	5
Age of Clients Served	Children (0–18)	13
	Adults (19–50)	16
	Older Adults (50+)	14
Service Provided	Assistive Technologies	8
	Related Services	12
	None	3

### Network analysis

We calculated five network metrics for each respondent. Mean values per type of respondent and range of scores across all respondents in each group are indicated in Table 3. Total possible score is indicated in the top row.

**Table 3.** Network metrics

	Mean Values *(Range)*				
Type of Respondent	In-Degree	Out-Degree	Weighted In-Degree	Weighted Out-Degree	Betweenness Centrality
Total Possible	19.0 *(0–19)*	19.0 *(0–19)*	76.0 *(0–76)*	76.0 *(0–76)*	1.00 *(0–1)*
Government Ministry (n = 4)	17.0 *(17–17)*	16.8 *(15–18)*	45.5 *(41–50)*	53.0 *(37–61)*	0.76 *(0.67–0.79)*
Service Delivery Organization* (n = 1)	18.0 (-)	17.0 (-)	52.0 (-)	56.0 (-)	0.79 (-)
Organizations of Persons with Disabilities (n = 6)	15.3 *(14–16)*	16.3 *(15–18)*	37.8 *(26–49)*	38.3 *(28–50)*	0.59 *(0.12–0.79)*
International NGO (n = 8)	14.9 *(9–18)*	14.4 *(6–18)*	35.9 *(14–43)*	31.2 *(8–53)*	0.58 *(0.24–0.79)*
All Organizations (n = 19)	15.6 *(9–18)*	15.6 *(6–18)*	39.4 *(14–50)*	57.5 *(8–61)*	0.63 *(0.12–0.79)*

*Only one service delivery organization is included; values are specific to that organization.

Overall, government ministries and service delivery organizations have the highest degree of connection to the network, with international NGOs having the lowest degree of connection to the network. While the number of inward and outward connections reported per organization are well-aligned, the strength of those connections may differ. In general, there are higher out-degree scores than in-degree scores. This suggests organizations report a higher degree of connection to other organizations than those same organizations report themselves. However, this is reversed for International NGOs, who had higher in-degree scores than out-degree scores.

**Figure 1.**
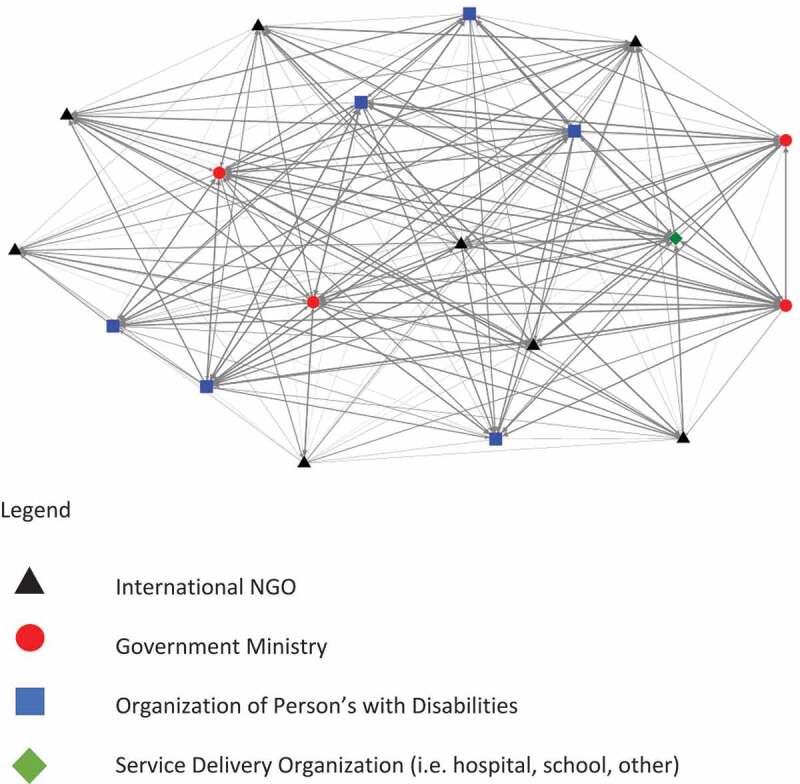
Graphical representation of network of assistive technology organizations in Malawi.

Figure 1 is a graphical representation of the network, with organizations of different types represented by different shape and colour nodes.

## Discussion

To our knowledge, this study represents the first network analysis of assistive technology organizations at a national level, and therefore provides a model for other researchers and policy developers wishing to understand the nature and strength of their national networks.

Our results suggest there is no single organization in Malawi which is central to the provision of assistive technology. In fact, there is a high degree of interconnectedness within and across the network, which spans different ministries and sectors. This is seen in both the network metrics and the graphical representation. Across the three ministries, and one coordinating body represented by the ministries, it is clear there is no single ministry which is seen as being central to assistive technology in Malawi.

This lack of a single point of centrality poses particular challenges, but also opportunities, for the development of policy. In many countries, including Malawi, it is conventional to have a single ministry responsible for leadership and delivery of programs and services in a particular area. For example, policy development guidance published by the Government of Malawi suggests the need to identify a single line ministry, who holds responsibility for policy development, implementation, and review [15].

Whilst recognizing the administrative value of a core or managing ministry, this is not incompatible with an opportunity to cultivate a distributed or collective leadership approach, where the responsibility for leadership and implementation is shared across ministries, reflecting the differing and legitimate interests of different sectors regarding assistive technology. Collective leadership has been documented to be effective at achieving meaningful change at the healthcare provision level and in developing policies where there are multiple stakeholders [16,17].

Our research also highlights the role of differing types of organizations, and their centrality to the delivery of assistive technology. We were interested to see that International NGOs, while playing a critical role in the funding and delivery of assistive technology, are not as central to the network as those organizations which represent people with disabilities, service delivery organizations, and ministries. This may mean that these organizations have more of a supportive than implementing role to play, at least in the case of Malawi. Furthermore, International NGOs appear to have fewer outward connections, than inward connections; suggesting there may be a high level of awareness of these organizations by national organizations, but the international NGOs do not have the same level of awareness of these national organizations. For more effective service delivery, international NGOs may therefore be better at providing funding to existing service delivery organizations and OPDs who have greater strength within the network, than seeking to provide services themselves.

In the course of this research, the Government of Malawi, in partnership with many of the stakeholders which were included as a part of this analysis, has been working towards substantive changes in assistive technology policy. Future research should look to identify whether these changes in policy have a resultant net effect on the nature and strength of the stakeholder network. The current research serves as a baseline evaluation of the network prior to the implementation of those changes.

### Limitations

We were unable to capture all relevant organizations working in Malawi in assistive technology, however our respondents do include those organizations which are the most engaged in the area. We were limited in our sample size, due to the nature of the assistive technology ecosystem in Malawi, which is currently limited in scope, with minimal national or donor driven funding. Furthermore, this represents only a cross-sectional view of the network, and existing relationships at the time of data collection. Finally, there may be response bias in the survey, where some organizations may have reported a higher degree of connection with other organizations to appear favorable in terms of their relationships with others and their role within the ecosystem. We were unfortunately not able to calculate measures of reliability to assess potential bias in the sample due to the small number of organizations working in the AT space in Malawi.

## Conclusions

The network of organizations delivering assistive technology services in Malawi is highly distributed, with no single organization or government ministry playing a central role. International NGOs are less connected to the network than other organizations operating within the country. A collective leadership approach which engages all relevant stakeholders and government ministries may the most effective in addressing the complexity of assistive technology service delivery, and the distribution of the network.

## Paper context

The landscape of assistive technology provision in Malawi has been explored in previous work, however has not identified the nature and strength between key stakeholders. In this research, we identified the relationships between these stakeholders to better understand the centrality of certain organizations. Understanding the nature and strengths of relationships allows the identification of organizations who can play a critical role in developing and implementing assistive technology policy.
